# Improved data acquisition efficiency for respiratory motion correction in coronary MRI

**DOI:** 10.1186/1532-429X-14-S1-P246

**Published:** 2012-02-01

**Authors:** Mehdi H Moghari, Sébastien Roujol, Markus Henningsson, Raymond H Chan, Susie Hong, Beth Goddu, Lois A Goepfert, Kraig V Kissinger, Warren J Manning, Reza Nezafat

**Affiliations:** 1 Harvard Medical School, Cambridge, MA, USA; 2 Medicine, Beth Isreal Deaconess Medical Center, Boston, MA, USA

## Summary

To investigate the performance of a novel algorithm for correcting respiratory-induced heart motion for whole-heart coronary MRI.

## Background

A right hemi-diaphragm (RHD) respiratory navigator is commonly used to suppress the respiratory motion of the heart in coronary MRI [1]. Typically, a small 5mm end expiratory gating window (GW) is used to gate data. While this technique successfully suppresses respiratory motion, it prolongs scan time due to navigator efficacies of only 30-50%. Increasing the GW to 15mm would increase the navigator efficiency. In this study, we present a novel respiratory motion correction algorithm which allows increasing the GW to 15mm.

## Methods

Whole-heart coronary MRI with isotropic 1.3mm^3^ resolution was acquired using partial Fourier and a navigator with 15mm GW on a 1.5T Philips CMR scanner from 10 healthy subjects (4 males; 29 ± 13 yr). The navigator RHD positions were used to sort the acquired k-space segments into 15 separate 1mm bins (based on their displacement). To compensate for the respiratory motion of the heart, all k-space segments acquired in each bin were assigned a 3D translation parameter. The 3D translation parameter was estimated using an iterative gradient descent optimization algorithm to correct the k-space segments such that the sharpness of the image, reconstructed using the corrected k-space segments, is maximized. The variance of the gradient of the image was used as the measure for the image sharpness [2]. For comparison, another whole-heart coronary MRI dataset was acquired using the same sequence and a navigator with a 5mm GW.

## Results

**Figure 1:**
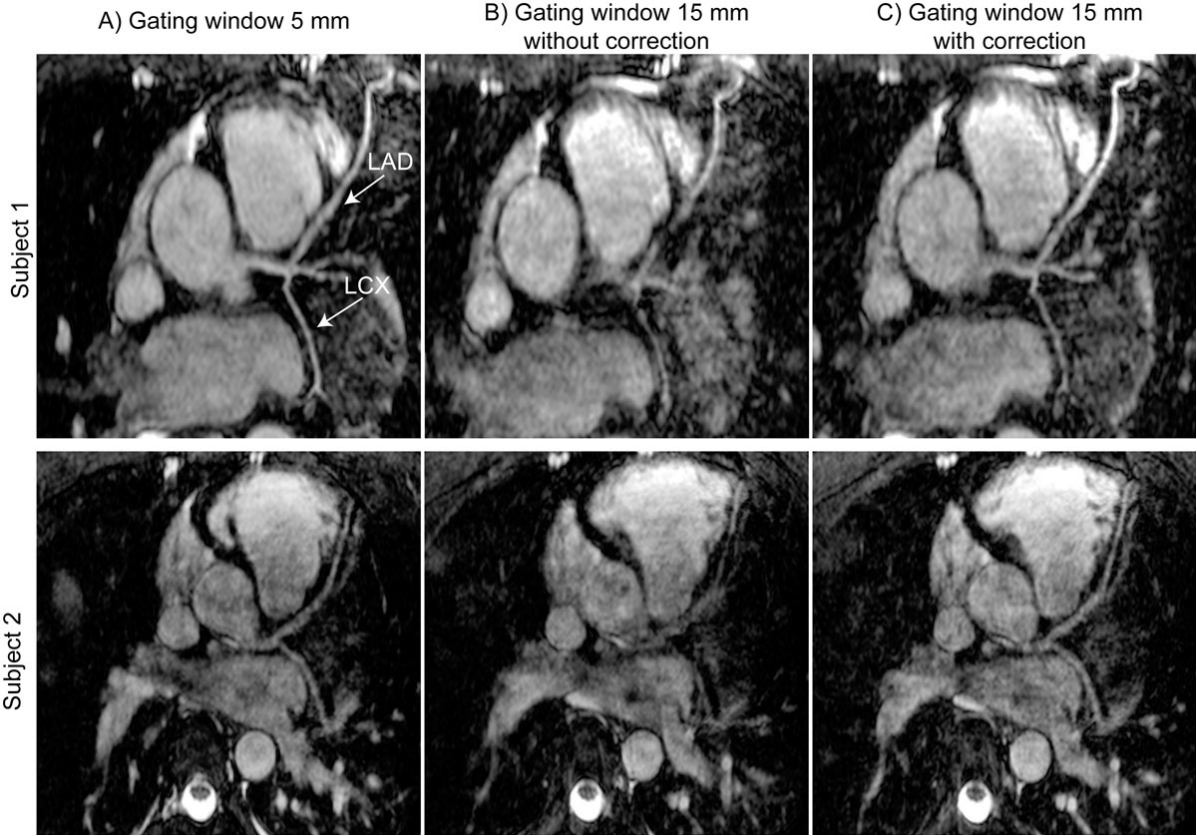
Reformatted whole-heart coronary MRI acquired from three healthy subjects using A) a right hemi-diaphragm (RHD) navigator with a 5 mm gating window; B) a RHD navigator with 15 mm gating window without correction and C) a RHD navigator with 15 mm gating window with correction. Arrows identify the left anterior descending (LAD) and left circumflex (LCX) arteries.

Fig.1 displays multi-planner reformatted images acquired using a 5mm GW (A), a 15mm GW without correction (B) and a 15mm GW with the proposed correction technique (C). There is an improvement between images acquired using 15mm GW with correction. The mean and standard deviation of scan acquisition time and efficiency as well as mean and standard deviation of normalized vessel sharpness of right coronary artery (RCA), left anterior descending (LAD) and left circumflex (LCX) arteries are shown in Table 1. However there is an improvement in the mean of the sharpness of LAD and LCX using the proposed technique compared to the reference, but it is not significantly different.

**Table 1:** Quantitative comparison between the whole-heart coronary MRI acquired with a navigator with a 5 mm gating window (GW), 15 mm GW without correction (noMC), and 15 mm GW with correction (MC) using the proposed algorithm. Higher values of sharpness are superior. All values are reported as mean ± standard deviation, and all statistically significant *p* values are in bold.

Parameter	5mm GW (REF)	15mm GW without Correction (noMC)	15mm GW with Correction (MC)	*p* value (n=10)		
	
				MC vs. REF	noMC vs. REF	MC vs. noMC
Imaging time (min.)	14 ± 5	7 ± 1	7 ± 1	**<0.002**	**<0.002**	-

Scan efficiency (%)	55 ± 13	92 ± 8	92 ± 8	**<0.001**	**<0.001**	-

RCA sharpness (mm^-1^)	0.51 ± 0.10	0.42 ± 0.17	0.49 ± 0.13	0.692	0.151	0.298

LAD sharpness (mm^-1^)	0.37 ± 0.15	0.29 ± 0.16	0.39 ± 0.04	0.683	0.271	0.079

LCX sharpness (mm^-1^)	0.33 ± 0.25	0.21± 0.19	0.38 ± 0.15	0.574	0.271	**<0.047**

## Conclusions

We present a novel retrospective RHD motion compensation algorithm for whole-heart coronary MRI that allows for widening GW and reduces scan acquisition time by a factor of 2.

## Funding

NIH.
